# Active time-reversal based phased array ultrasound system for accurate focusing through biological tissue layers

**DOI:** 10.1186/s11671-025-04331-6

**Published:** 2025-09-01

**Authors:** HaoSheng Xu, ShaoHui Yang, Qi Lai, XueMei Gao, WeiJuan Chen, XiaoJing He

**Affiliations:** 1 Department of Radiology, The Second Affiliated Hospital of Chongqing Medical University, Chongqing, China; 2 Department of Radiology, Chongqing Health Center for Women and Children, Chongqing, China; 3 Department of Radiology, Women and Children’s Hospital of Chongqing Medical University, Chongqing, China; 4 NHC Key Laboratory of Birth Defects and Reproductive Health, Chongqing, China

**Keywords:** Ultrasonic phased array, Delay resolution, Phase calibration, FPGA, Time reversal method

## Abstract

Ultrasonic phased array technology enables flexible and controllable beamforming through precise phase delay control of individual array elements in the transducer, facilitating dynamic focusing, beam steering, and beamforming. This study presents a 64-channel system achieving 1 ns delay resolution using FPGA-based phase-locked loops. Through systematic testing and calibration of the delay error in the phased array transmission driving system, the actual delay error was successfully controlled within 1 ns. Furthermore, to address the focal shift issue in multi-layer soft tissues, this research implemented active time reversal and phase compensation methods for focal shift correction. Experimental results demonstrate that the proposed system not only exhibits excellent driving and phase modulation capabilities but also effectively reduces tissue-induced focal shift.

## Introduction

High-Intensity Focused Ultrasound (HIFU) has emerged as a clinically validated, minimally invasive therapy that induces localized tissue ablation through two primary mechanisms: (1) thermal-mediated protein coagulation and (2) non-thermal mechanical disruption [1]. The technology enables precise spatial control, generating confined regions of coagulative necrosis (> 55 °C) at target depths while sparing surrounding tissues. Compared to alternative thermal therapies (e.g., radiofrequency/microwave ablation), HIFU offers superior temperature regulation and penetration depth in soft tissues, making it particularly valuable for treating solid tumors and other deep-seated pathologies.

Current HIFU systems utilize two primary transducer configurations: bowl-shaped and phased-array. The bowl-shaped focused transducer, characterized by its simple structure and fixed focal length, adjusts the focal position through mechanical movement. This technology has been widely implemented in clinical practice [2, 3]. In contrast, phased-array focused transducer utilizes software-controlled focusing mechanisms, offering several advantages including flexible focusing patterns, precise and rapid acoustic field control, beam steering capability, and multi-focus generation for enhanced ablation area and rate. These advanced characteristics endow phased-array technology with particular promise for treating solid tumor-related pathologies [4]. Jing et al. [5] employed the k-space method to correct transcranial ultrasound beam focusing, demonstrating that this approach enables precise targeting while completing treatment planning computations within minutes. Li et al. [6] proposed a method to enhance the steering range of HIFU phased arrays through array tilting, which effectively expands the focal steering capability and significantly increases the treatment volume. All these tissue or cranial treatments encounter a common challenge: the significant impact of multilayered media's nonlinear and heterogeneous characteristics on focusing efficacy. In addition, the delay resolution is also an important parameter that determines the focusing accuracy [7].

To address these issues, this study employs an active time-reversal method to correct focal shifts caused by the nonlinear and heterogeneous characteristics of multilayered tissues, proposing an innovative dual-delay approach that combines coarse and fine delay mechanisms to significantly improve delay resolution while enhancing echo sampling resolution through cross-correlation and curve-fitting interpolation methods. The experimental setup used two-layer porcine tissue (fat and muscle) as the propagation medium, with hydrophone measurements characterizing the steered focusing acoustic field of the ultrasound phased array.

## Materials and methods

### Principle of beam steering in phased-array ultrasound focusing

**Fig. 1:**
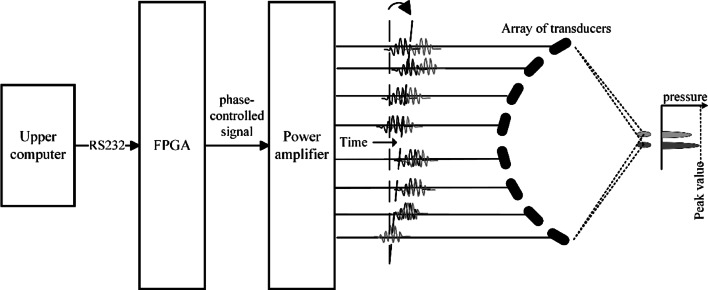
Schematic diagram of beam steering and focusing principles in phased-array systems

The excitation of each array element in the transducer software precisely controls, enabling the generation of time-delay-induced phase shifts ($\Delta \varphi =2\pi f\Delta t$) generate steerable wavefronts. This controlled phase variation creates coherent wavefronts that undergo constructive and destructive interference in the acoustic field, resulting in precise beam focusing and steering. The beamforming process obeys the Huygens-Fresnel principle by modeling each element as a secondary wavelet source, where each array element acts as a point source, and the superposition of these wavefronts determines the resultant beam direction and focal characteristics. The reception process represents the inverse of the transmission mechanism. Due to varying propagation paths, each array element receives echo signals from the target point at different times with distinct phase shifts. This spatial diversity necessitates precise time delay compensation and weighted summation during signal reception. The delay-and-sum beamforming algorithm enables flexible control over the synthetic aperture characteristics through manipulation of phase and amplitude parameters across array elements. This sophisticated control mechanism facilitates various operational modes, including conventional focusing, dynamic focusing, synthetic aperture imaging, and multi-beam formation [6]. The schematic diagram of phased-array focusing and beam steering as showed in Fig. 1. It is mainly composed of phase control system, power amplifier circuit, impedance matching circuit and phased-array transducer. In the HIFU phase control system, the host computer transmits the delay time of each channel to the FPGA via a serial interface. The FPGA then synchronizes the excitation signals of all channels using its internal clock to achieve phase control.

### Transducer geometry

**Fig. 2:**
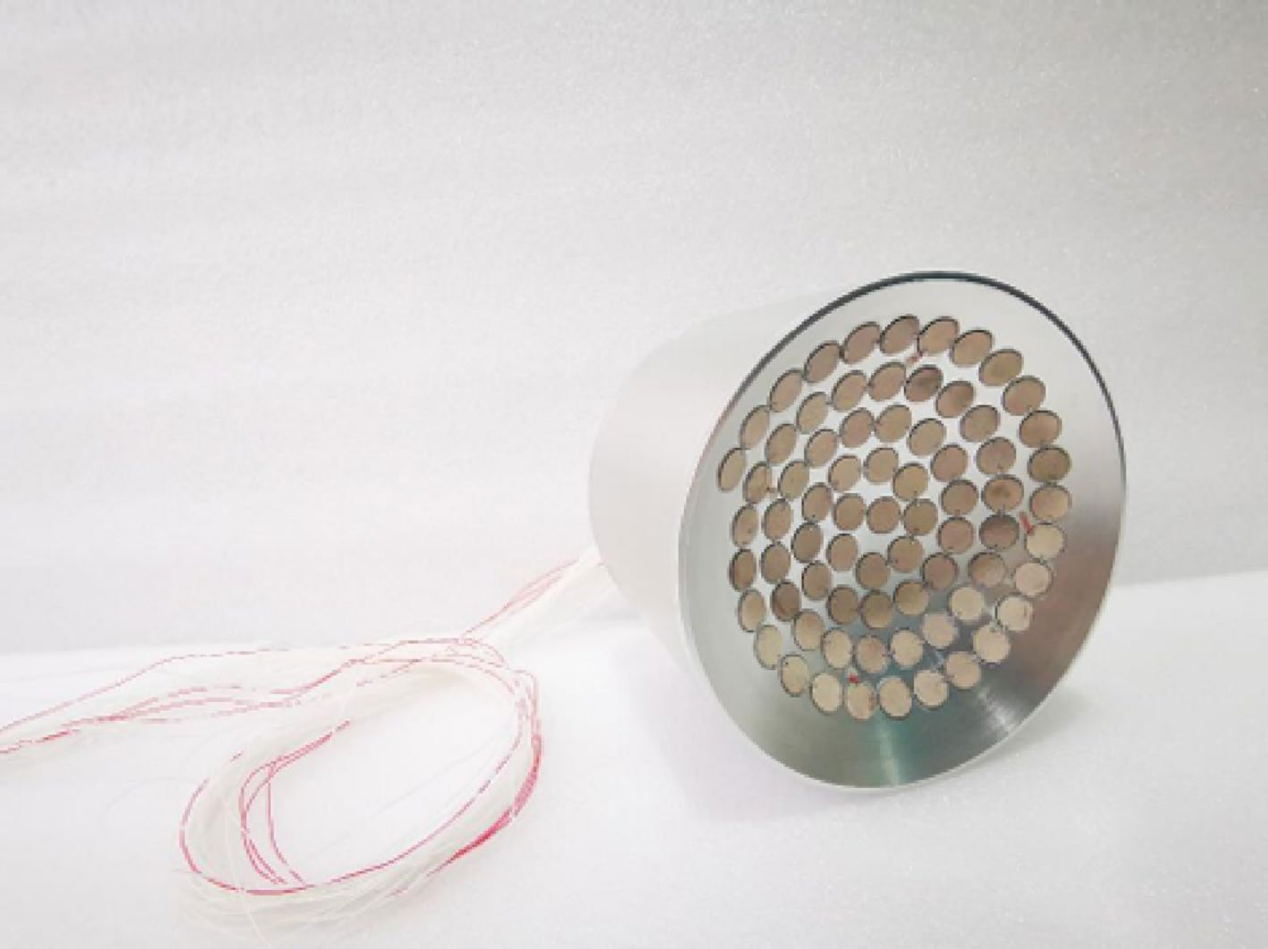
Physical diagram of 64 channel phased array transducer

The transducer employed in this study is a 64-element Archimedean spiral phased array fabricated from 1 to 3 piezoelectric composite material. This composite structure embeds piezoelectric ceramic pillars within a polymer matrix, combining the high piezoelectric performance of PZT with the polymer's advantageous characteristics including low acoustic impedance, reduced dielectric constant, and enhanced flexibility. The array demonstrates superior beam steering capabilities with effective sidelobe suppression, which is critical for therapeutic applications requiring precise focal control [8]. Figure 2 shows the physical implementation of this transducer, with the following key geometric parameters: element diameter (2r) = 13 mm, aperture diameter (D) = 150 mm, focal length (F) = 120 mm, and operating frequency (f) = 0.905 MHz.

The concave spherical transducer represents a section of a spherical surface, where the physical focal point coincides with the geometric center of the sphere when no time delays are applied. Considering the transducer's center as the origin $(\text{0,0},0)$ and the sphere's radius as r, the physical focal point coordinates are $(0,0,r)$ in the three-dimensional Cartesian coordinate system. For a given sound velocity c, let the spatial coordinates of the nth array element be ${(x}_{n},{y}_{n},{z}_{n})$ and the desired focal point coordinates be $(a,b,c)$. The acoustic path length (${F}_{n}$) between the nth array element and the designated focal point can be calculated using the three-dimensional Euclidean distance formula [9]:

1 $${F}_{n}={\left[{\left(a-{x}_{n}\right)}^{2}+{\left(b-{y}_{n}\right)}^{2}+{\left(c-{z}_{n}\right)}^{2}\right]}^{1/2}$$

After calculating the acoustic path difference between each element relative to the set focus, the acoustic path difference of each element is subtracted from the maximum value:

2 $$\Delta {F}_{n}=\text{max}\left({F}_{n}\right)-{F}_{n}$$

Once you have the relative distance, divide it by the speed of sound:

3 $${k}_{n}=\frac{\Delta {F}_{n}}{c}\times {10}^{9}$$

The time-reversal focusing method is related to the invariance of wave propagation equations under time reversal in lossless, fluid media. In soft tissues, the low attenuation coefficient within clinically used frequency ranges ensures the validity of the time-reversal invariance assumption. If ${P}_{r0}\left(ri, t\right)$ represents the pressure field recorded on the transducer array (where ${r}_{i}$ denotes the spatial position of array element ${E}_{i}$) from a pulsed point source at location $r0$,the time-reversal mirror inverts the signal to obtain ${P}_{r0}(r,T-t)$, where $T$ is the total delay time required for acoustic wave focusing. The time-reversed waves converge toward their source, effectively implementing a spatiotemporal matched filter that adapts to the propagation transfer function in heterogeneous media [10]. In fact, this method has been proven to optimally retrieve the source position of acoustic signals propagating along the optimal path in ${P}_{r0}(ri,T-t)$. According to the acoustic reciprocity theorem, even in heterogeneous media, the positions of transmitting and receiving transducers can be interchanged without altering the acoustic signal information. That is, the diffraction impulse response ${h}_{r0}\left(ri, t\right)$ measured at point $ri$ from a source at r0 is equivalent to the response ${h}_{r0}\left(r0, t\right)$ measured at r0 from a source at $ri$:

4 $${h}_{r0}({r}_{i},t)={h}_{ri}({r}_{0},t)$$

Assuming the piezoelectric and inverse piezoelectric effects of the transducer array are identical, then:

5 $${h}_{i}^{ae}(t)={h}_{i}^{ea}(t)$$

where $ae$ represents the piezoelectric process and $ea$ the inverse piezoelectric process. Consequently, after a complete time-reversal process (recording and transmitting signals), the total acoustic field received at $r0$ equals:

6 $$ A(r_{0} ,t) = \sum\limits_{{i = 1}}^{N} {h_{{ri}} (r_{0} ,t)*h_{i}^{{ea}} (t)*h_{{r0}} (r_{i} ,T - t)*h_{i}^{{ae}} (T - t)} $$

where $N$ is the number of transducer elements. According to Eq. (6), each element's contribution can be symmetrically expressed as:

7 $${a}_{i}\left({r}_{0},t\right)={h}_{ri}\left({r}_{0},t\right)*{h}_{r0}\left({r}_{i},T-t\right)*{h}_{i}^{ea}\left(t\right)*{h}_{i}^{ae}\left(T-t\right)$$

These consecutive convolutions implement matched filtering of diffraction impulse responses and transducer responses. Based on matched filter theory, the acoustic waves from each element reach their maximum at point $r0$ at time $T.$

### FPGA-based transmission delay design methodology

Delay resolution is the most critical parameter in phased array technology, determining the system's imaging resolution and error sidelobes. Dusa et al. [11] implemented a 3.125 ns receive delay resolution using dedicated integrated chips at 40 MHz sampling frequency, while proposing a modular architecture for complete beamforming that enables 1.25 ns minimum delay resolution per channel. Tang et al. [12] proposed a novel polyphase CIC interpolation filter design, achieving 1 ns delay resolution at 100 MHz sampling frequency using an improved 10 × polyphase interpolation CIC filter. Yuhang et al. [13] utilized two PLL resources in FPGA to double and phase-shift a 50 MHz clock signal, generating eight 250 MHz square wave channels with 45° phase differences, achieving a simulated delay resolution of 0.5 ns. Excessively high delay resolution imposes significant pressure on hardware circuits, causing substantial deviations between actual errors and theoretical values. For practical therapeutic applications, 1 ns delay resolution is entirely sufficient. Digital delay techniques such as sampling delay and dedicated chip delay suffer from high costs and difficulties in modification and integration. Software delay has gradually become the primary delay control method due to its strong flexibility, versatility, and portability. Considering the main frequency of the FPGA chip used in this phased array system and implementation feasibility, the simpler PLL-based method was selected for high delay resolution design. This approach achieves an effective balance between delay precision and implementation complexity while maintaining reasonable power consumption.

**Fig. 3:**
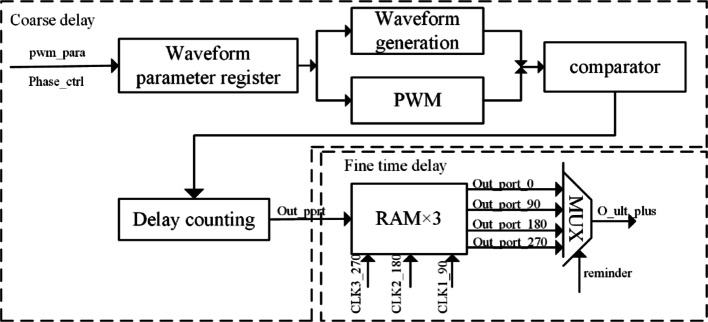
Conceptual illustration of the delay module design

The proposed delay module architecture adopts a coarse–fine hybrid delay mechanism, as illustrated in Fig. 3. The system utilizes FPGA-integrated Multi-Clock Management Modules (MCMM) to generate precise clock signals through accurate frequency multiplication and phase shifting, achieving high delay precision at relatively low operating frequencies.

The coarse delay section utilizes a 250 MHz clock signal obtained by multiplying a 50 MHz crystal oscillator input by a factor of 5 using a phase-locked loop (PLL). Based on the 250 MHz clock frequency counting, the delay value is an integer multiple of the clock cycle, yielding a resolution of 4 ns. Due to the limited operating frequency of the FPGA, a combined approach of fine delay and coarse delay is adopted to further refine the excitation waveform output from the coarse delay stage.

**Fig. 4:**
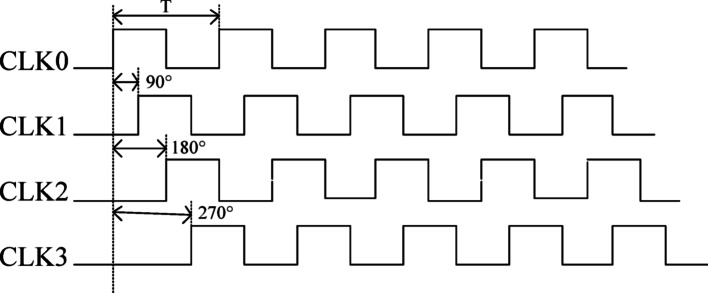
PLL phase shift timing sequence

By employing the PLL to multiply the 50 MHz crystal input by 5 and generate three phase-shifted clock signals with 90° phase differences, the 250 MHz clock corresponds to a 4 ns cycle, resulting in a 1 ns time difference between two clock signals with 90° phase shift. This enables a theoretical fine delay resolution of 1 ns in addition to the coarse delay. The schematic diagram of the PLL frequency multiplication for fine delay implementation is shown in Fig. 4.

### Phase correction methodology for tissue-induced errors

The focal point of concave spherical focusing transducers exhibits significant displacement from the geometric center when propagating through multi-layer soft tissues, primarily due to heterogeneous media and nonlinear effects. This focal shift becomes particularly pronounced when encountering high-density media such as skull or rib structures during acoustic propagation. Furthermore, research has demonstrated that nonlinear effects in concave spherical focusing systems induce additional focal displacement, with the focal point migrating away from the transducer as signal amplitude increases [14]. The acoustic parameters of each tissue layer are listed in Table 1, with data sourced from the IT'IS Foundation in Zurich, Switzerland.

**Table 1:** Media acoustic parameters table

Medium	Speed of sound (m/s)	Density (kg/m^3^)	Attenuation coefficient (Np/m)	Nonlinear parameter (B/A)
Water	1500	1000	0.025	5.0
Skin	1624	1109	21.254	7.0
Fat	1478	911	4.360	10.0
Muscle	1588	1090	7.100	7.1

**Fig. 5:**
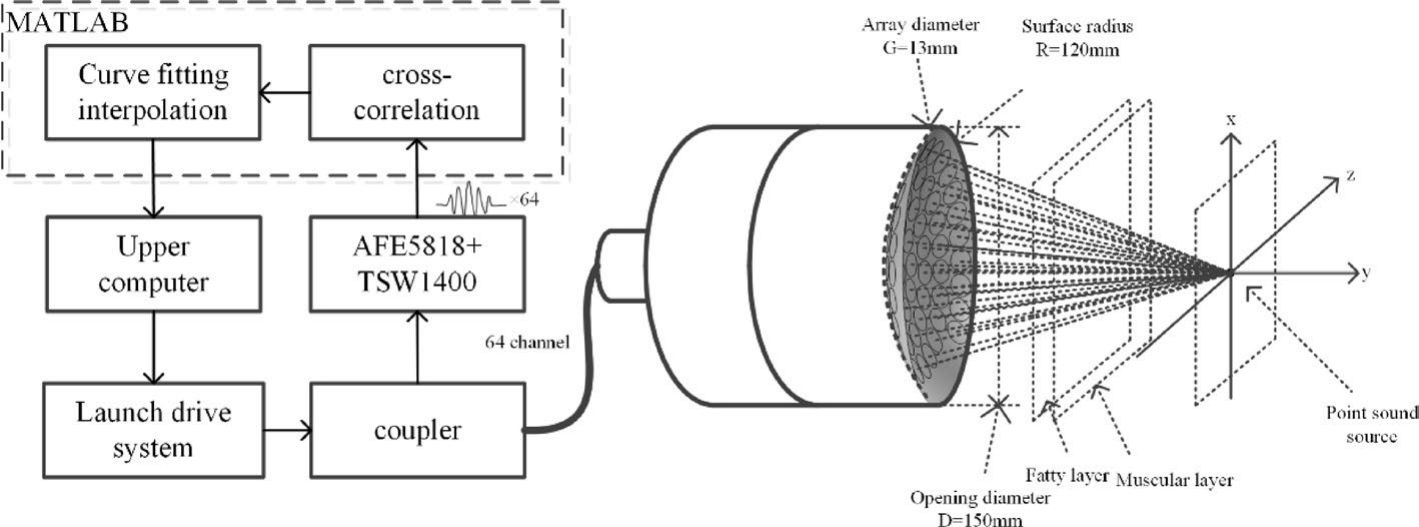
The time reversal method excessively centralizes the correction focus

Time-reversal methodology has emerged as an effective solution for compensating focal shifts in heterogeneous media [15]. Extensive studies by domestic and international researchers have validated the efficacy of time-reversal techniques in compensating focal displacements under both linear and nonlinear conditions [15, 16]. Building upon these findings, this study implements an active time-reversal method to compensate for focal shifts occurring through multi-layer tissues and aqueous media. The specific method is shown in Fig. 5.

In the forward propagation phase, the wavefront emitted from a point source propagates through the medium and is subsequently detected by one or more receivers. The detected signals at each receiver undergo temporal inversion and are retransmitted from their respective positions, causing the retransmitted signals to converge at the original source location. This receiver array constitutes the time-reversal mirror (TRM), which in ultrasound systems typically comprises a 64-element phased array transducer capable of sampling, time-reversing, and retransmitting the incident acoustic field [17].

The time-reversal (TR) process can be conceptualized as a wave energy concentration method that exploits wave back propagation to focus energy at specific spatiotemporal locations. This technique fundamentally relies on two physical principles: spatial reciprocity and time-reversal invariance of the linear wave equation. In heterogeneous media, TR focusing demonstrates unique capabilities.

The accuracy of TR-based tissue-induced focal shift correction critically depends on the precision and efficiency of delay estimation algorithms. This study implements a normalized cross-correlation (NCC) method for echo signal delay estimation. The NCC algorithm extracts a data segment (window) from the reference echo signal and searches for the most matching pattern in the shifted echo signal by computing the correlation coefficient (pattern matching function) at each search step [18]. The NCC between the reference $s_1[n]$ and the offset $ss_2[n]$ window is defined as:

8 $${R}_{NCC}\left[\tau \right]=\frac{{\sum }_{i=r}^{r+N-1}({s}_{1}[i]\cdot {s}_{2}[i+\tau ])}{\sqrt{{\sum }_{i=r}^{r+N-1}{s}_{1}^{2}[i]\cdot {\sum }_{i=r}^{r+N-1}{s}_{2}^{2}[i+\tau ]}}$$

In the normalized cross-correlation (NCC) algorithm, r represents the origin of the reference window, N denotes the window length, and τ indicates the search lag (i.e., the offset between the reference window and the shifted window). The numerator in the NCC equation represents the unnormalized cross-correlation between $s_1[n]$ and $s_2[n]$, while the denominator normalizes this correlation by accounting for the energy of both windows. This normalization process provides significant advantages by compensating for local variations in standard deviation and window mean values [19].

**Fig. 6:**
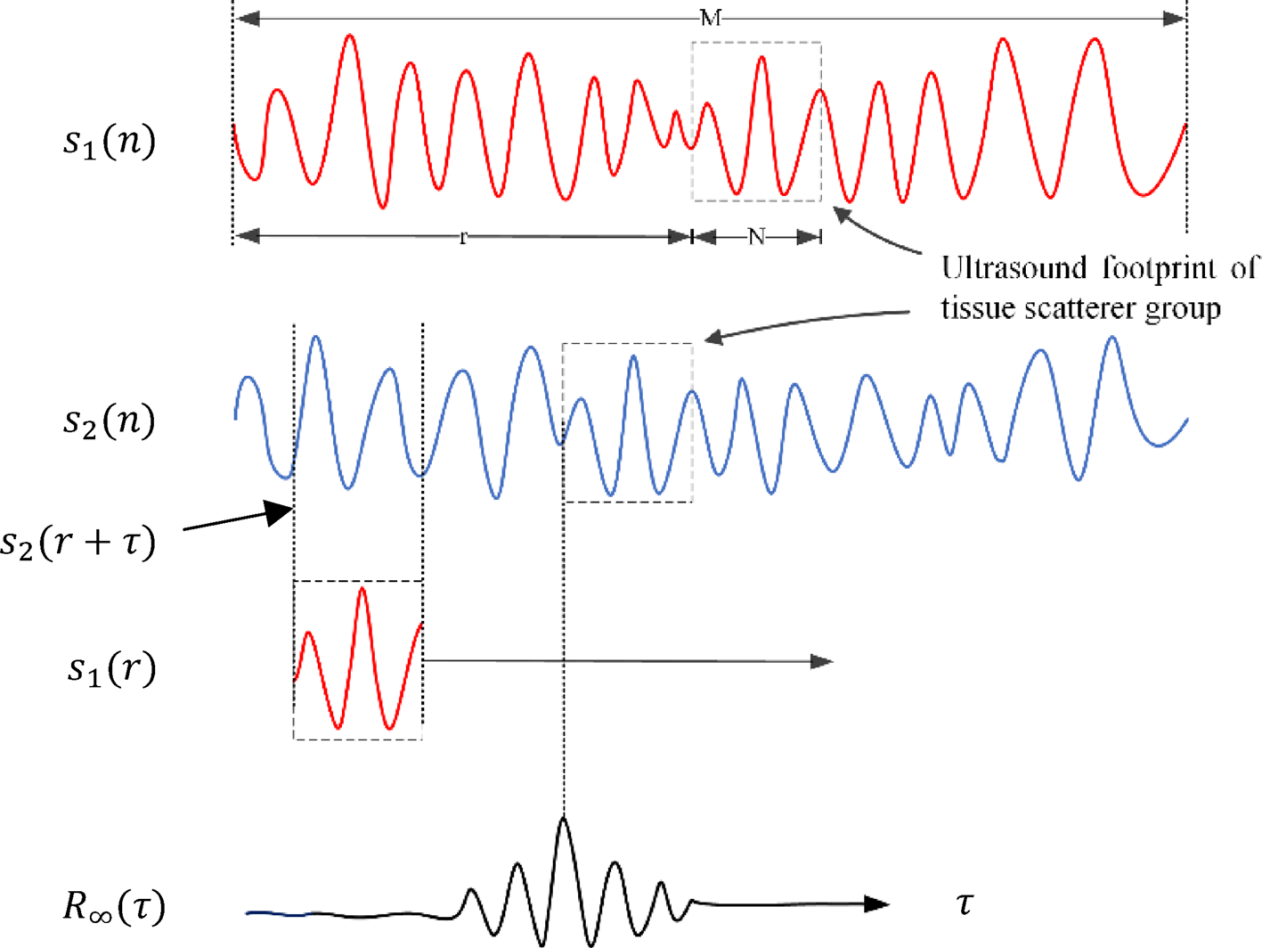
A schematic illustration of a correlation coefficient based motion estimation method. A reference window is compared with a shifted window to find the location where correlation coefficient between them is maximum. The location of maximum correlation coefficient corresponds to the time-shift between the two windows

The time shift between the reference window and the shifted window is determined by the τ value that maximizes the NCC coefficient (Eq. 8). This maximum correlation point corresponds to the optimal temporal alignment between the two signal segments. Figure 6 illustrates the general workflow of motion estimation based on correlation coefficients.

In practical implementation, the estimation of time delays using discrete-time ultrasonic echo signals is inherently limited to integer multiples of the sampling interval. This discrete-time approach may introduce significant estimation bias. However, when the sampling rate exceeds the Nyquist rate, interpolation techniques can achieve time delay estimation accuracy comparable to continuous-time signal processing.

**Fig. 7:**
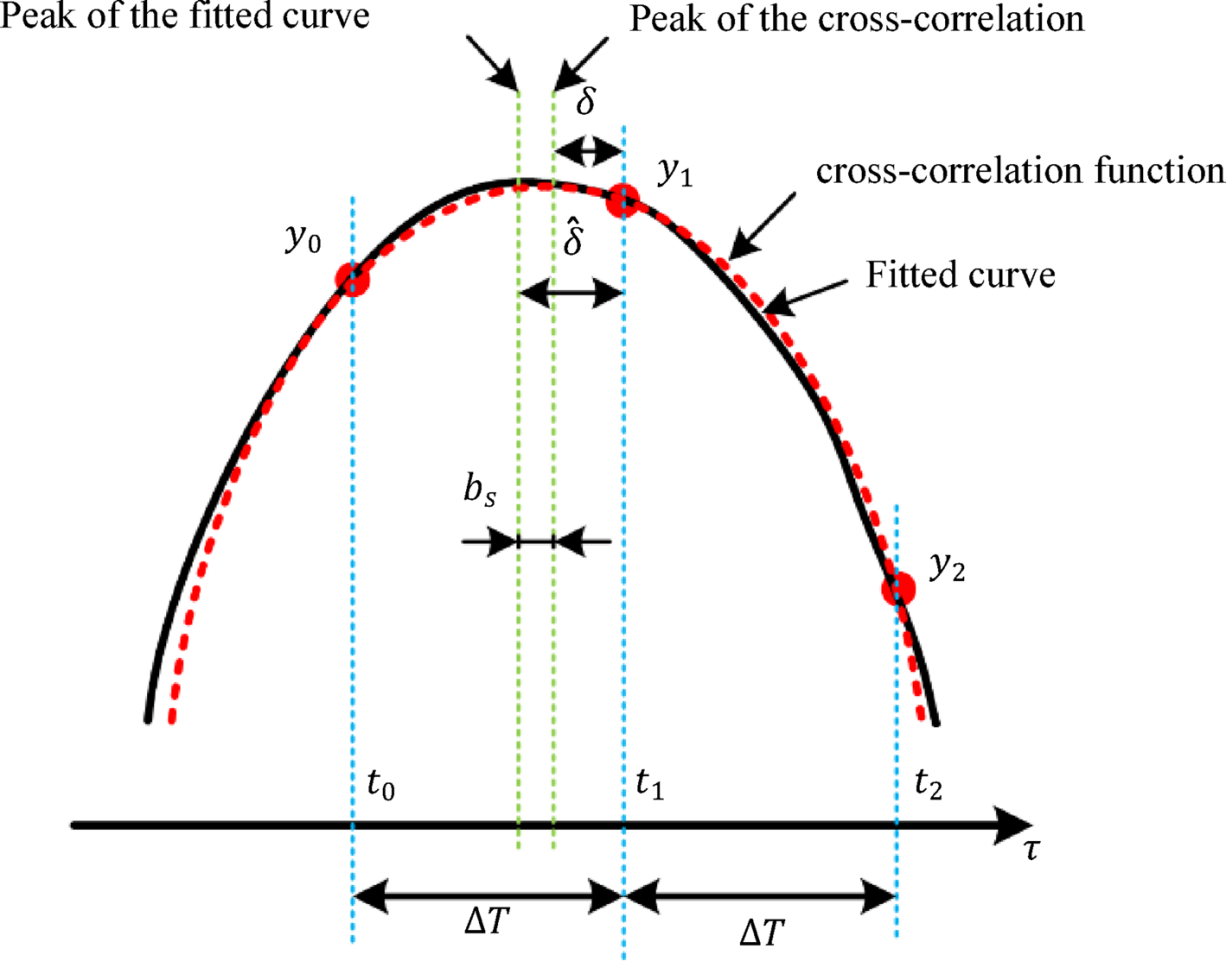
Diagram of the curve fitting to the three sampling points (*y*0, *y*1, and *y*2) around the cross-correlation peak. The dashed line represents the continuous time cross correlation, and the solid line represents the fitting curve

The echo acquisition system in this study operates at a sampling frequency of 50 MHz, yielding an initial delay estimation resolution of 4 ns. This resolution mismatch with the transmission system's delay resolution (1 ns) could potentially introduce measurement errors. To address this limitation, we implement a three-point peak interpolation method with 40 interpolation points following cross-correlation analysis, effectively enhancing the delay estimation resolution to 1 ns, thereby matching the transmission system's resolution. Figure 7 illustrates the curve-fitting interpolation process of the cross-correlation function.

The pre-interpolation delay estimation is constrained to integer multiples of the sampling interval, while post-interpolation resolution improves proportionally with the number of interpolation points between samples. The cosine curve fitting method applies a cosine function to three adjacent sampling points ($y_0$, $y_1$, and $y_2$) near the peak of the cross-correlation function, defined by:

9 $$\widehat{\delta }=\frac{\alpha }{\beta }$$

α and β are defined as:

10 $$\alpha ={\text{cos}}^{-1}\left(\frac{{y}_{0}+{y}_{2}}{{2y}_{1}}\right)$$

11 $$\beta ={\text{tan}}^{-1}\left(\frac{{y}_{0}-{y}_{2}}{{2y}_{1}\text{sin}(\alpha}\right)$$

Without curve fitting or interpolation, time delay estimation would be constrained to the sampling point with maximum cross-correlation. However, when considering continuous-time signal processing, the peak of the cross-correlation function would be offset by $\delta$ from $t$. By fitting a curve to several sampling points surrounding the cross-correlation peak (specifically $y_0$, $y_1$, and $y_2$), the precise location of the maximum $\hat{\delta}$ can be analytically determined.

### Experimental setup and configuration

**Fig. 8:**
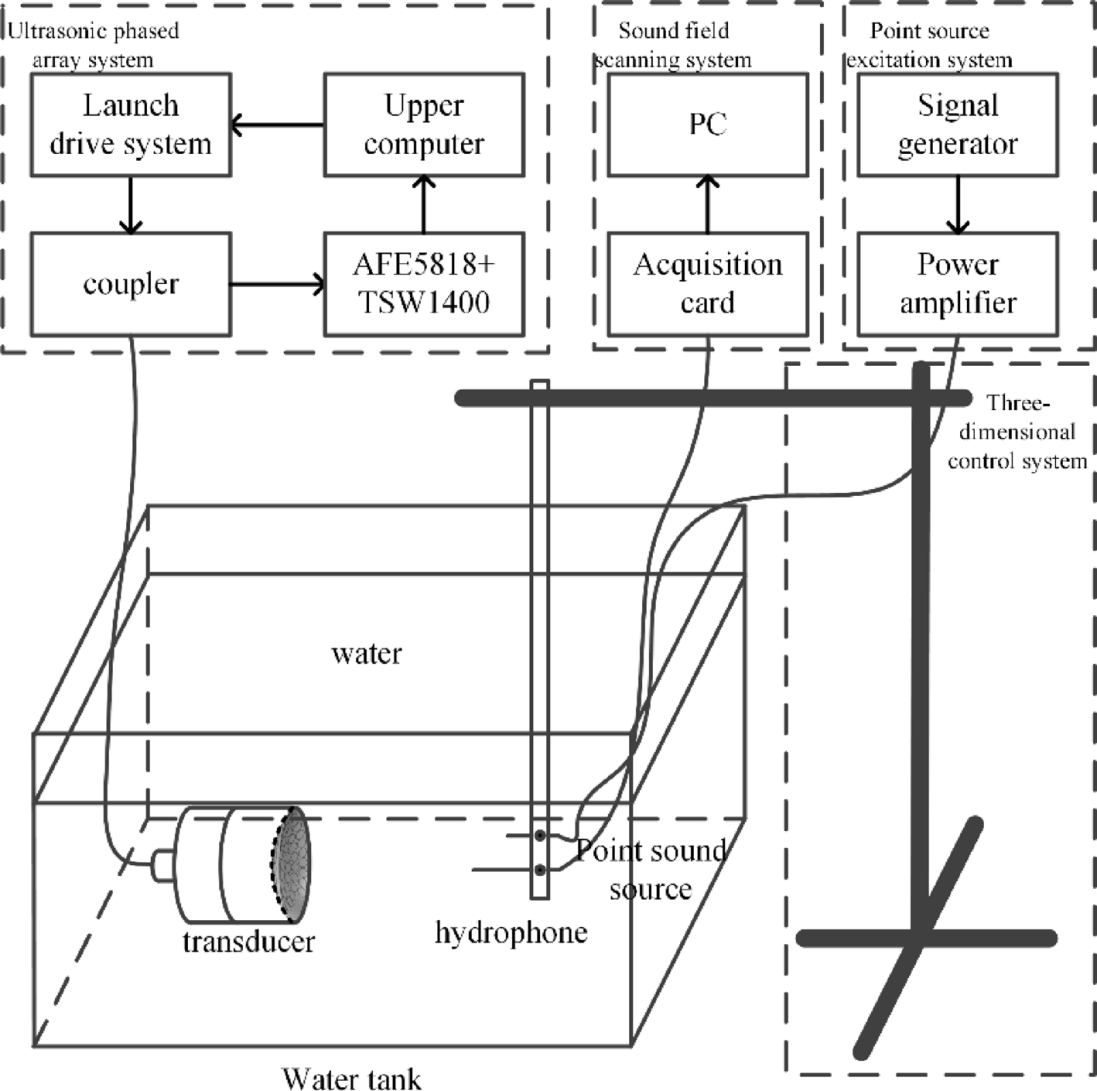
Experimental platform

The experimental platform was designed to systematically evaluate the performance of the phased-array system through comprehensive sound field measurements. As shown in Fig. 8, the setup integrates four major components: an ultrasonic phased-array system, a sound field scanning system, a point sound source excitation system, and a precision motion control system. The ultrasonic phased-array system features a 64-channel programmable array driver controlled through a custom LabVIEW interface, combined with an AFE5818 analog front-end and TSW1400 digital processor for echo reception and signal processing. For sound field characterization, an ONDA HGL-0085 hydrophone with 0.2 mm active diameter was mounted on a motorized 3-axis positioning stage offering 0.01 mm resolution. The hydrophone signals were acquired through a 14-bit PCIe digitizer sampling at 100 MS/s, with spatial scanning controlled by a custom MATLAB algorithm. The point source excitation system consisted of an arbitrary waveform generator (20 MHz bandwidth) driving a power amplifier (50 dB gain) to stimulate a 10 MHz focused transducer producing a 0.8 × 2 mm focal zone.

The measurement protocol established a Cartesian coordinate system centered on the array aperture, with the hydrophone scanned in 0.1 mm increments across a 50 × 50 × 50 mm volume. Each measurement point recorded full waveform data for subsequent analysis including pressure amplitude, spatial intensity distribution, focal zone dimensions, and sidelobe characteristics. This integrated experimental configuration enabled precise quantification of array performance parameters while maintaining rigorous measurement standards essential for reliable phased-array characterization. The combination of high-resolution motion control, sensitive acoustic detection, and comprehensive data processing provided a robust platform for evaluating delay resolution effects on beamforming quality.

## Results and discussion

### Comparison of FPGA simulation and actual results

**Fig. 9:**
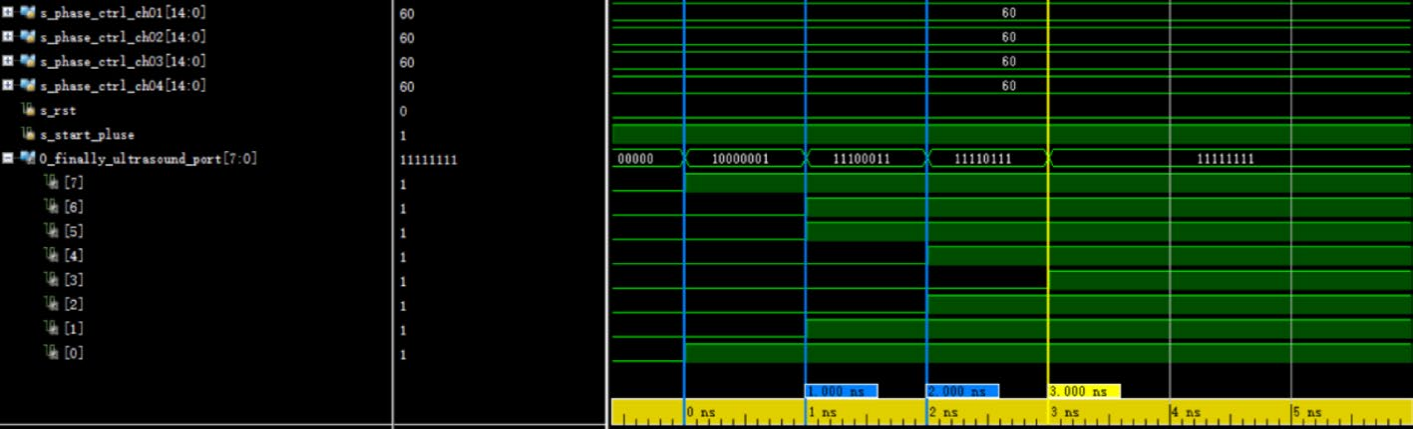
Delay simulation of frequency doubling and phase shifting

In order to verify the correctness of the system design, Vivado simulator is used for simulation test. The simulation results are shown in Fig. 9, which reflects the output timing sequence of 8 pulse excitation signals. The coarse delay time of each channel in the test file is set to 60 ns, and the remainder of the first four channels is set to 0,1,2,3, respectively. The values set correspond to the first four digits of the O_finally_ultrsound_port signal. At the same time, the data bit width of each channel phase control signal is 15 bits, and the dynamic range after multiplying by 4 ns period is 0–13.1 us. The desired pulse excitation signal is generated at the synchronization start signal and the reset signal for the high level and low level respectively.

It can be seen from the figure that the difference of channels 1, 2, 3, and 4 is 1 ns, which proves that the theoretical delay resolution reaches 1 ns, thus verifying the correctness of the FPGA delay module design and conforming to the proposed parameter indexes.

**Fig. 10:**
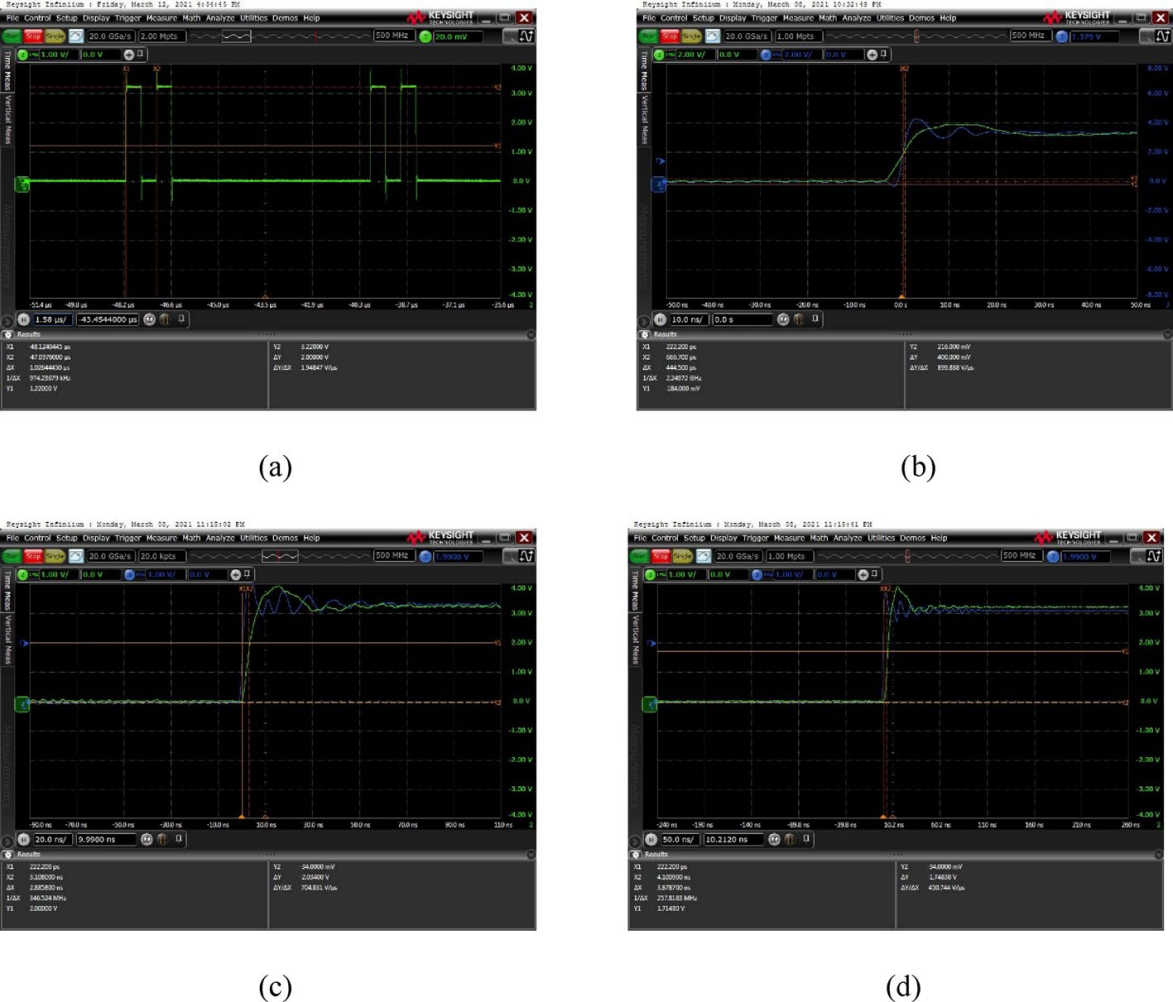
**a** Ultrasonic frequency and modulation waveform test results; **b** 1 and 2 channel delay resolution test results; **c** 1 and 3 channel delay resolution test results; **d** 1 and 4 channel delay Resolution test result

Since the underlying control program for each FPGA channel is identical, only four adjacent channels were tested. The delay values for channels 1, 2, 3, and 4 were set to 0, 1, 2, and 3, respectively. The test results of ultrasound frequency and modulated waveforms are shown in Fig. 10a. The delay resolution test results between channels 1 and 2 are presented in Fig. 10b, between channels 1 and 3 in Fig. 10c, and between channels 1 and 4 in Fig. 10d

Figure 10a shows the test results with an output frequency of 1 MHz, modulation frequency of 2000 Hz, and output amplitude of 3.22 V. The on-board measured delay error values for Fig. 10b, c, and d are summarized in Table 2.

**Table 2:** FPGA board level test results

Channel (refer to Channel 1)	Theoretical delay value (ns)	Actual delay value (ns)	Error value (ns)
2-channel	1	0.444	− 0.556
3-channel	2	2.385	0.385
4-channel	3	3.878	0.878

It can be seen from Table 2 that compared with the actual delay value and the theoretical delay value, the error values of the first three channels are all within 1 ns, and the overall delay resolution is within 2 ns, which conforms to the parameter indexes set by the subject. This design only uses 3 dual-port RAM to achieve the actual 2 ns delay resolution, and has the advantages of small error, less resource consumption, strong scalability, and the use of residual judgment form to solve the problem of cross-clock domain skillfully.

### Actual results of physical field focusing deflection

**Fig. 11:**
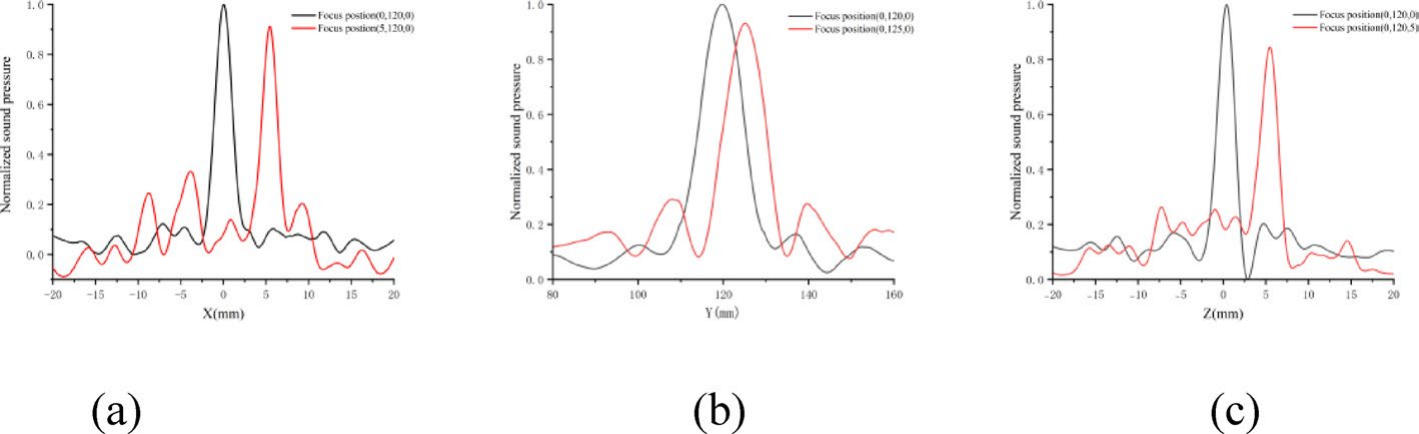
**a** 5 mm normalized sound pressure distribution of X-axis geometric focusing and deflection; **b** Y-axis geometric focusing and deflection 5 mm normalized sound pressure distribution; **c** Z-axis geometric focusing and deflection 5 mm normalized sound pressure distribution

The transmission drive system drives the 64-channel phased array transducer to emit ultrasonic waves for sound field scanning. Through the upper computer, the 64-channel delay is set to 0, the ultrasonic frequency is 905 kHz, the power is 1W, the duty cycle is 50%, and the modulation frequency is 1000 Hz. After setting the relevant parameters, the sound field x, Y and z axis distribution is measured by scanning the hydrophone. The sound field distribution after normalization is shown in Fig. 11, in which Fig. (A), (B) and (C) respectively represent the normalized sound pressure distribution above and below the X axis, Y acoustic axis and left and right direction of the Z axis.

Figure 11a–c show the acoustic pressure distribution curves of our time-reversal method focusing at preset coordinate points (0, 120, 0) and (5, 125, 5) in water conditions. The black curves represent (0, 120, 0), which is the transducer's natural focal center point, while the red curves represent (5, 125, 5), indicating a 5 mm deflection in all three directions. The results demonstrate that maximum acoustic pressure achieves good focusing at (0, 120, 0). For the 5 mm deflection experiments in X, Y, and Z directions, the maximum acoustic pressure occurs at (5.2, 125.1, 5.3), with focusing position errors within 0.3 mm, fully proving the accuracy and feasibility of our research. Through three repeated experiments, we confirmed that the errors in X, Y, and Z directions are all within 0.3 mm, further demonstrating the stability of our research system.

### Time reversal method corrects focus error

**Fig. 12:**
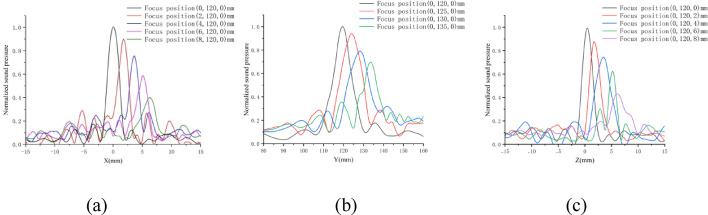
**a** displays the experimental data of the steered acoustic field in the positive X-axis direction (vertical) within the tissue, **b** presents the steered acoustic field data in the positive Y-axis direction (acoustic axis, away from the transducer), and **c** shows the steered acoustic field data in the positive Z-axis direction (lateral) within the tissue

The acoustic beam steering test results in the previous section indicate that focal position may deviate in aquatic environments due to equipment errors and other factors. Meanwhile, the nonlinearity of layered tissues can also cause focal shifts. The time-reversal method was employed to calibrate and compensate for both types of errors. After performing delay estimation on the echo data, 40 interpolation points were generated from 3 sample points near the peak of the cross-correlation curve for curve fitting. The sampling frequency matched the theoretical delay resolution of the excitation system. Using the estimated delay values, the transducers were re-excited by the driving system to emit ultrasound through tissues for acoustic field scanning. The normalized acoustic field distribution after multilayer tissue time-reversal calibration for focal shift correction is shown in Fig. 12. The experimental data from 10 repeated trials of beam steering in the positive X-, Y-, and Z-axis directions are presented in Tables 3, 4, and 5, respectively.

**Table 3:** The experimental data from 9 beam-steering tests along the X-axis direction in tissues (data are presented as median)

Preset focal offset in positive X-axis direction (mm)	0	2	4	6	8
Actual focal position (mm)	0.2[0.1, 0.3]	1.7[1.5, 1.9]	3.5[3.3, 3.7]	5.1[4.9, 5.3]	6.3[6.1, 6.6]
Focal sound pressure (p/p0)	1	0.88[0.88, 0.92]	0.76[0.73, 0.79]	0.59[0.53, 0.61]	0.40[0.34, 0.42]

**Table 4:** The experimental data from 9 beam-steering tests along the Y-axis direction in tissues (data are presented as median)

Preset focal offset in positive Y-axis direction (mm)	120	125	130	135
Actual focal position (mm)	119.5 [119.3, 119.8]	124.1[123.6, 124.9]	128.6[128.0, 129.1]	133.2[132.5, 133.9]
Focal sound pressure (p/p0)	1	0.91[0.85, 0.93]	0.79[0.75, 0.81]	0.65[0.61, 0.73]

**Table 5:** The experimental data from 9 beam-steering tests along the Z-axis direction in tissues (data are presented as median)

Preset focal offset in positive Z-axis direction (mm)	0	2	4	6	8
Actual focal position (mm)	0.3 [0.2, 0.5]	1.6[1.4, 1.8]	3.5[3.4, 3.8]	5.2[4.7, 5.5]	6.1[5.7, 6.4]
Focal sound pressure (p/p0)	1	0.87[0.84, 0.90]	0.74[0.71, 0.77]	0.58[0.53, 0.62]	0.39[0.35, 0.42]

The results show that the time-reversal method can achieve accurate focusing at preset positions through multiple biological tissue layers. In the Y-axis (acoustic axis) direction, when offsetting from the geometric focus (non-steered focus) at 120 mm by 5 mm, 10 mm, and 15 mm away from the transducer, nine repeated experiments yielded median actual focus positions of 119.5 mm, 124.1 mm, 128.6 mm, and 133.2 mm, with focusing errors of 0.5 mm, 0.9 mm, 1.4 mm, and 1.8 mm respectively. For the X (vertical) and Z (lateral) axes, with the Y-axis fixed at 120 mm, when steering from 0 to 8 mm in 2 mm increments, nine experiments showed median X-axis errors of 0.2 mm, 1.7 mm, 3.5 mm, 5.1 mm, and 6.3 mm, and Z-axis errors of 0.5 mm, 1.6 mm, 3.5 mm, 5.2 mm, and 6.1 mm.

The Y-axis demonstrates excellent focusing performance, maintaining errors within 2 mm even at 15 mm offset (1.8 mm error), meeting HIFU treatment requirements in human tissue. The X and Z axes also maintain good focusing accuracy, enabling dynamic 3D steering. Their comparable accuracy is likely due to the transducer's near-symmetrical spatial distribution about the Y-axis plane. In addition, focal pressure decreases with increasing offset distance—dropping to 40% of the geometric focus pressure at 8 mm offset in X/Z axes, while the Y-axis shows better pressure maintenance at 65% at 15 mm offset. This suggests focal pressure distribution after steering may become an interesting research direction.

In summary, our study demonstrates that time-reversal focusing through multiple tissue layers can achieve accurate focusing at preset positions. Despite nonlinear tissue effects, thermal effects, and inherent system hardware interference factors, it maintains focusing accuracy within small error ranges, meeting HIFU treatment requirements in human tissues.

## Conclusion

Conventional HIFU focusing transducers employ relatively simple focusing mechanisms that cannot alter focal positions without physically moving the transducer. Currently, phased-array ultrasound focusing has emerged as a major research focus, particularly for biomedical applications. Phased-array systems can drive multi-element focusing transducers, electronically control focal positions through software-configurable excitation delays to achieve dynamic focusing automatically, and regulate energy distribution in focal regions to better ablate pathological tissues, thereby improving treatment efficacy and efficiency. When conventional delay-based focusing methods are applied in phased-array systems through multi-layered structures, significant focal shifts occur relative to preset positions due to strong nonlinear effects from high-intensity focusing, thermal effects, and errors from material processing and circuitry. These deviations become more pronounced with increasing steering distances.

 In this study, our optimized phased-array system employs an active time-reversal method to precisely determine preset focal delays. The ultrasound energy maintains excellent focusing through multi-layered biological tissues, with maximum focal errors below 2 mm—meeting the ≤ 2 mm precision requirement for non-invasive brain tumor ablation. Three-dimensional focal accuracy satisfies HIFU therapeutic standards.Future phased-array ultrasound focusing may replace traditional single-element spherical focusing systems in biomedicine, particularly for HIFU therapy. Extensive research on precise cranial modulation is already underway, and accurate focal control in abdominal applications will be crucial for clinical translation. Additionally, temperature rise in target regions requires special consideration. Under identical irradiation conditions, deeper tissues exhibit smaller temperature increases, slower heating rates, and more dispersed energy distribution, resulting in progressively reduced effective heating areas (> ~ 40 °C). Variations in acoustic (sound speed, attenuation coefficient) and thermal (thermal conductivity, specific heat capacity) parameters among tissue types also lead to different focal temperatures under identical conditions.

Future studies should address the therapeutic impact of focal temperature variations and develop phased-array strategies for optimal energy control and distribution in focal regions—a key direction for our subsequent research. For clinical implementation of phased-array systems, two major challenges must be resolved: focal positioning accuracy and energy distribution patterns. This study provides essential theoretical and experimental support for advancing phased-array ultrasound systems toward clinical applications.

## Data Availability

Data will be made available on request.
